# Characterization of dystroglycan binding in adhesion of human induced pluripotent stem cells to laminin-511 E8 fragment

**DOI:** 10.1038/s41598-019-49669-x

**Published:** 2019-09-10

**Authors:** Yumika Sugawara, Keisuke Hamada, Yuji Yamada, Jun Kumai, Motoi Kanagawa, Kazuhiro Kobayashi, Tatsushi Toda, Yoichi Negishi, Fumihiko Katagiri, Kentaro Hozumi, Motoyoshi Nomizu, Yamato Kikkawa

**Affiliations:** 1 Department of Clinical Biochemistry, Tokyo University of Pharmacy and Life Sciences, Tokyo, 192-0392 Japan; 2 Division of Molecular Brain Science, Kobe University Graduate School of Medicine, Kobe, Hyogo 650-0017 Japan; 3 Department of Neurology, Graduate School of Medicine, The University of Tokyo, Tokyo, 113-0033 Japan; 4 Department of Drug Delivery and Molecular Biopharmaceutics, Tokyo University of Pharmacy and Life Sciences, Tokyo, 192-0392 Japan

**Keywords:** Peptides, Biomaterials - proteins, Extracellular matrix

## Abstract

Human induced pluripotent stem cells (hiPSCs) grow indefinitely in culture and have the potential to regenerate various tissues. In the development of cell culture systems, a fragment of laminin-511 (LM511-E8) was found to improve the proliferation of stem cells. The adhesion of undifferentiated cells to LM511-E8 is mainly mediated through integrin α6β1. However, the involvement of non-integrin receptors remains unknown in stem cell culture using LM511-E8. Here, we show that dystroglycan (DG) is strongly expressed in hiPSCs. The fully glycosylated DG is functionally active for laminin binding, and although it has been suggested that LM511-E8 lacks DG binding sites, the fragment does weakly bind to DG. We further identified the DG binding sequence in LM511-E8, using synthetic peptides, of which, hE8A5-20 (human laminin α5 2688–2699: KTLPQLLAKLSI) derived from the laminin coiled-coil domain, exhibited DG binding affinity and cell adhesion activity. Deletion and mutation studies show that LLAKLSI is the active core sequence of hE8A5-20, and that, K2696 is a critical amino acid for DG binding. We further demonstrated that hiPSCs adhere to hE8A5-20-conjugated chitosan matrices. The amino acid sequence of DG binding peptides would be useful to design substrata for culture system of undifferentiated and differentiated stem cells.

## Introduction

Human induced pluripotent stem cells (hiPSCs) are promising cells for regenerative medicine, similar to human embryonic stem cells (hESCs). These cells have an infinite proliferative potential and capacity for differentiation into all cell types of the body. They are prospective cell sources for applications such as transplantation therapy and drug discovery. The ability to stably expand the stem cells is a fundamental technical requirement for these applications. Stem cells were originally maintained in complex culture systems, comprising a mouse feeder cell layer, media containing foetal bovine serum, an extracellular matrix-rich environment for cell adhesion, and soluble growth factors^1^. The batch-to-batch variability of biological materials limits scalability. Many groups have tried to optimize xeno-free culture conditions for stem cells^2^. Of these approaches, modification of the culture substrata has improved the proliferation and expansion of hESCs and hiPSCs. The culture substrata can be divided into synthetic substrata, including Synthemax and PMEDSAH, and recombinant protein-derivative culture substrata including laminins and vitronectin^3–6^. However, the optimization of culture substrata for the maintenance of stem cells continues to be a challenge. Therefore, further improvement in the culture systems is required for the efficient and stable expansion of stem cells.

A recombinant fragment of laminin-511 (LM511-E8), commercially available as iMatrix-511, is currently used as a culture substrate in combination with various defined medium systems^7,8^. Laminins are a family of glycoproteins found in basement membranes^9^. They are composed of three disulfide-linked subunits, the α, β, and γ chains. Up to now, five α, three β and three γ chains have been characterized, and 19 different laminin heterotrimeric isoforms have been identified in various tissues and cell culture media. Of these isoforms, laminin-511 (LM511; α5, β1, γ1) is widely distributed in the basement membranes of foetal and adult tissues^10^. LM511 is also present in blastocysts and contacts the inner cell mass that is the origin of ES cells^11^. Lack of the α5 chain does not influence the proliferation of the inner cell mass, suggesting that LM511 is not involved in stemness during early development^12^. However, because LM511 is useful for stem cell culture, LM511 is still considered to be involved in maintaining the pluripotency of the inner cell mass.

The interaction of cells with laminins is mediated by various receptors, including integrins and non-integrins^9^. Intact LM511 is bound by integrin α3β1, α6β1, α6β4, α7β1, dystroglycan (DG), and Lutheran/basal cell adhesion molecule (Lu/B-CAM)^13–17^. Cell adhesion to α5-containing laminins is mediated through the binding of integrin α3β1/α6β1 and Lu/B-CAM to the LG1-3 modules, and the preferential binding of dystroglycan to sites in the LG4-5 modules^15,16,18^. Consistent with previous studies, LM511-E8, which contains the LG1-3 modules, exhibits potent cell attachment activity, mediated through integrin α3β1, α6β1, α6β4, and α7β1^19,20^. In human stem cells, integrin α6β1 is a major isoform at cell surfaces^7,21,22^. The fragment containing the integrin α6β1-binding site, enables superior adhesion of single cell-dissociated cultures of hESCs and hiPSCs^7^. Therefore, it is well-known that the adhesion of hiPSCs to LM511-E8 is mainly mediated through integrin α6β1. However, it is unknown whether non-integrin receptors are expressed in stem cells and play roles in pluripotency and differentiation of the cells, cultured on laminins and their fragments.

Defined media and substrata are required for the culture system in order to effectively exploit stem cells for clinical use. In this study, in order to define the adhesion of cultured hiPSCs to LM511-E8, we analyzed the expression of DG in hiPSCs, examined the biochemical properties of DG in hiPSCs cultured on LM511-E8, and characterised the DG binding sequences in LM511-E8 using synthetic peptides. We also explored the possibility that DG-binding peptides could serve as culture substrata. Our results provide useful information to design substrata for culture system of undifferentiated and differentiated stem cells.

## Results

### Analysis of DG expression in hiPSCs

**Figure 1:**
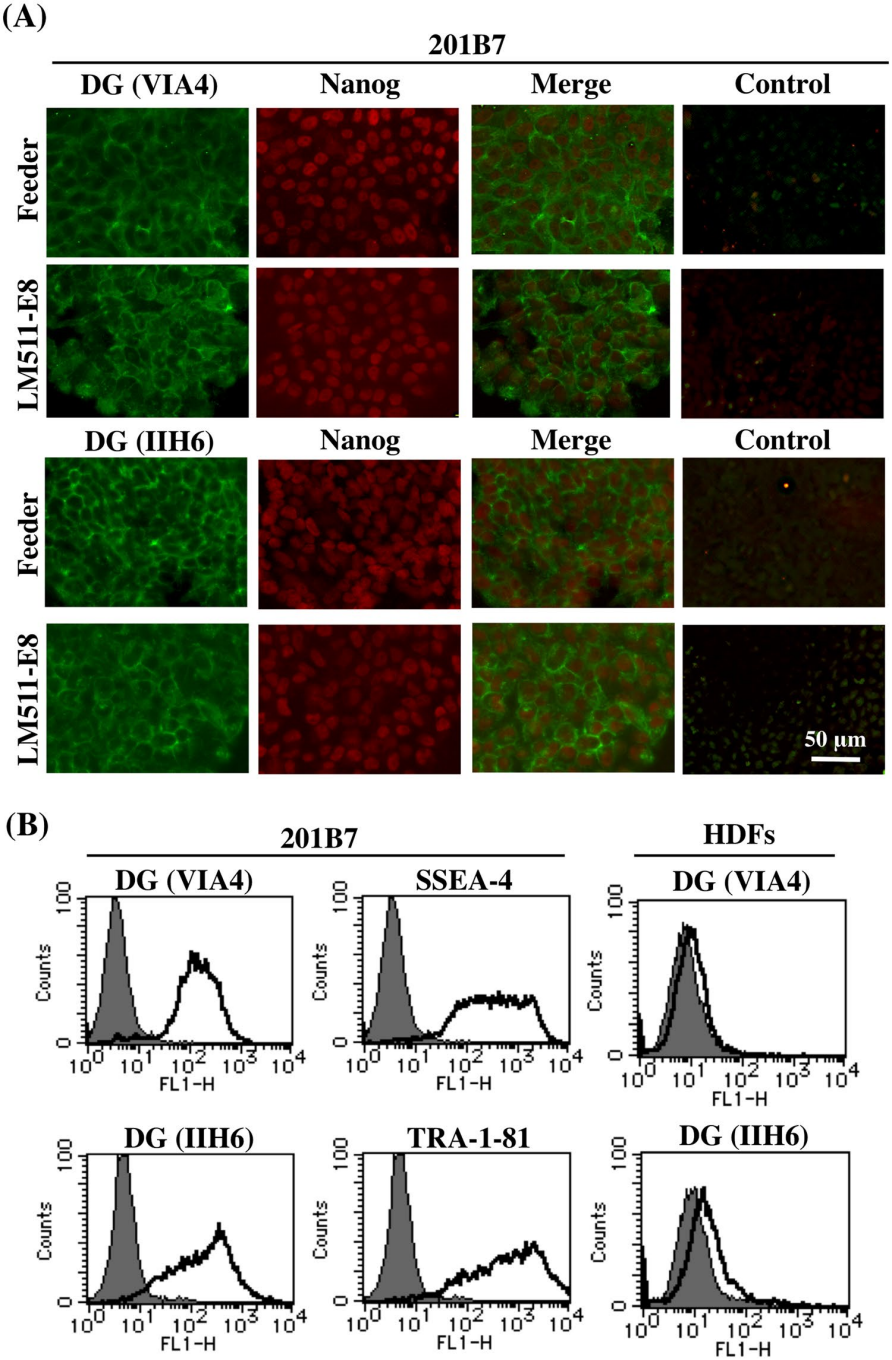
DG is expressed by hiPSCs. (**A**) Immunostaining for DG and Nanog in hiPSCs (201B7), cultured on feeder cells or LM511-E8. Colonies were doubly stained with antibodies (VIA4 and IIH6) to DG (green) and Nanog (red). Images of DG and Nanog staining are merged. Control is without primary antibodies. DG is detected in hiPSCs regardless of culture methods. (**B**) Flow cytometric analysis of DG and expression of two stem cell markers in hiPSCs (201B7) and human dermal fibroblasts (HDFs). The expression of DG (VIA4 and IIH6), SSEA-4, and TRA-1-81 are shown as solid lines. Grey fill indicates control IgG (mouse IgG, upper panel; mouse IgM, lower panel). DG is highly expressed in the hiPSCs but not HDFs.

Dystroglycan is a non-integrin receptor that is mainly expressed in muscle and nervous system^23^. Previous studies have demonstrated DG expression in many other cell types, including mouse ES cells^24–26^. Therefore, we examined the expression of DG in hiPSCs. Two hiPSC clones were used in this study: 201B7 and 454E2, derived from human dermal fibroblasts and dental pulp cells, respectively^27,28^. Immunocytochemistry was first performed to analyse DG expression in hiPSCs cultured in different systems (Fig. 1A). Two monoclonal antibodies, VIA4 and IIH6, were used for the immunostaining. Both antibodies recognized the fully glycosylated α-DG^29^. IIH6 also detects the laminin binding glycoepitope on α-DG, and hypoglycosylation results in the absence of epitopes, for this antibody. VIA4 antibody staining showed that DG was uniformly detected in 201B7 colonies, cultured on either feeder cells or LM511-E8. DG positive cells usually expressed Nanog, which is an undifferentiated state maker. We next performed flow cytometric analysis using hiPSCs cultured on LM511-E8. The results showed that 201B7 cells mostly expressed DG that was recognized by both antibodies (Fig. 1B). The results indicate that hiPSCs express the laminin binding glycoepitope on α-DG. Similar results were observed in 454E2 cells (Fig. S1). However, human dermal fibroblasts (HDFs), which are of hiPSC origin, did not express DG.

**Figure 2:**
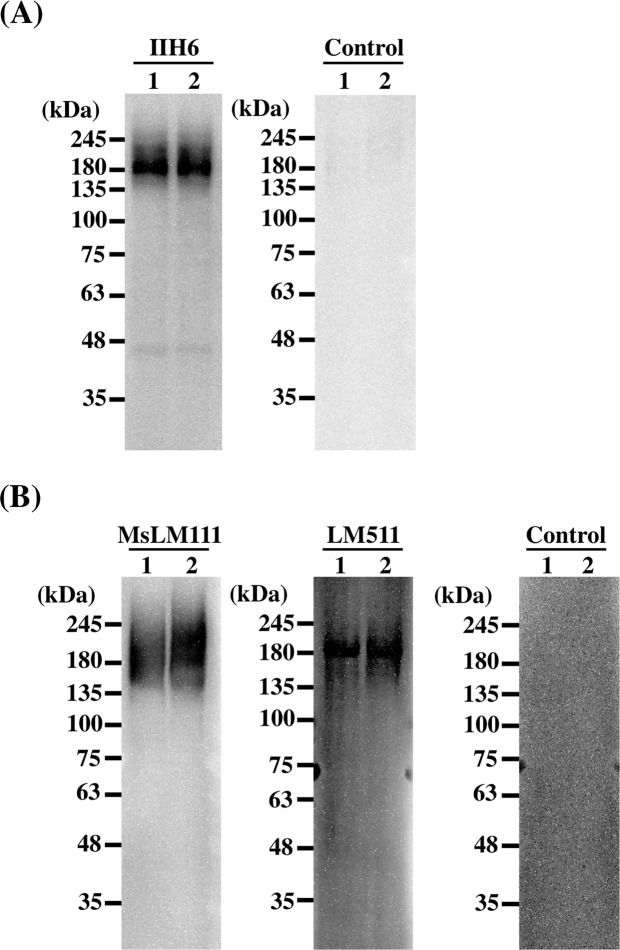
Biochemical analysis of DG expressed in hiPSCs. DG was enriched from 201B7 (lane 1) and 454E2 (lane 2) cell lysates using wheat germ agglutinin (WGA) agarose. The WGA complexes were separated on a 5–20% gradient gel under reducing conditions. (**A**) Immunoblotting of anti-α-DG monoclonal antibody (IIH6, left panel). Control is without primary antibody (right panel). α-DG migrated at 170–240 kDa. Molecular mass standards are indicated. (**B**) Laminin overlay assay. Mouse EHS laminin-111 (MsLM111) and human laminin-511 (LM511) were overlaid on the membrane (left and centre panels); control is without laminins (right panel). The overlaid MsLM111 and LM511 bound at 150–250 kDa and 170–200 kDa, respectively.

Additionally, we performed biochemical analysis of DG expression in hiPSCs. After SDS-PAGE separation, α-DG retains its laminin binding property on PVDF membranes^30^. We performed immunoblotting and laminin overlay assay to examine if DG expressed in hiPSCs, exhibits binding affinity. DG was partially purified from the cell lysates using wheat germ agglutinin (WGA) agarose as described in previous study^31^. Immunoblotting using IIH6 antibody showed that the highly glycosylated α-DG, from both hiPSC clones, migrated broadly at 170–240 kDa (Fig. 2). The laminin overlay assay showed that α-DG of hiPSCs was functionally active. Because MsLM111 overlay assay was more sensitive than the immunoblotting, the band of α-DG migrated more broadly at 150–250 kDa. Moreover, we examined the binding of LM511 that is used for stem cell culture. Although, LM511 bound to α-DG on the membrane, the bands were narrower than those of MsLM111.

### The binding of DG to LM511-E8

**Figure 3:**
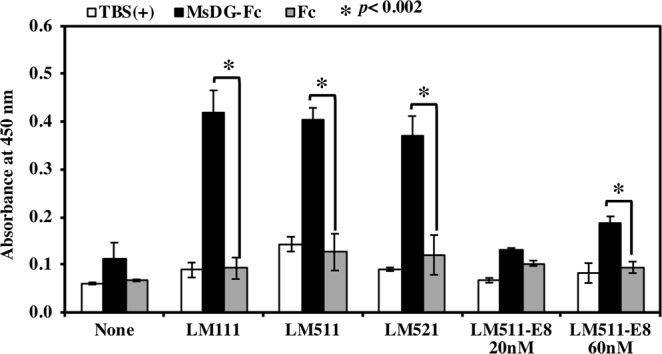
Solid-phase binding assays of MsDG-Fc to immobilized recombinant laminins. The conditioned media containing MsDG-Fc or control Fc were prepared as described in Methods. Ninety six-well ELISA plates were coated with recombinant proteins at 20 nM (LM111, LM511, LM521, and LM511-E8) or 60 nM (LM511-E8). After blocking, the wells were incubated with conditioned media containing MsDG-Fc or Fc at room temperature for 1 hour. Bound MsDG-Fc was detected with anti-human IgG Fc antibody.

LM511-E8 now serves as a cell culture substratum that maintains the pluripotency of hESCs and hiPSCs^7^. High expression of DG in hiPSCs allowed us to examine the binding of the DG to LM511-E8. Solid phase binding assays to LM511-E8 were performed using a soluble recombinant DG (MsDG-Fc) composed of mouse α-DG fused to human IgG_1_ Fc^32^. Because purified MsDG-Fc was unstable, we used conditioned medium containing MsDG-Fc, for the solid phase binding assays. Conditioned medium, containing Fc, was used as control. LM-111 was used as a positive control in the overlay assay. We first compared the DG binding affinity between α1 and α5 chains, using LM-111 and -511. Both molecules were recognized to similar extents by MsDG-Fc (Fig. 3). Although, the substitution of laminin β1 with laminin β2 in LM-521, promotes survival of human pluripotent stem cells without ROCK inhibitor^33^, the β2 chain did not influence the binding of DG. MsDG-Fc bound to the plate coated with 60 nM of LM-511-E8, however, DG failed to bind to it at 20 nM concentration. These results indicate that although domains of LM-511, other than the E8 region, harbour the major DG binding sites, LM511-E8 does contain DG binding sites.

### DG binding activity of human laminin α5 chain peptides

**Figure 4:**
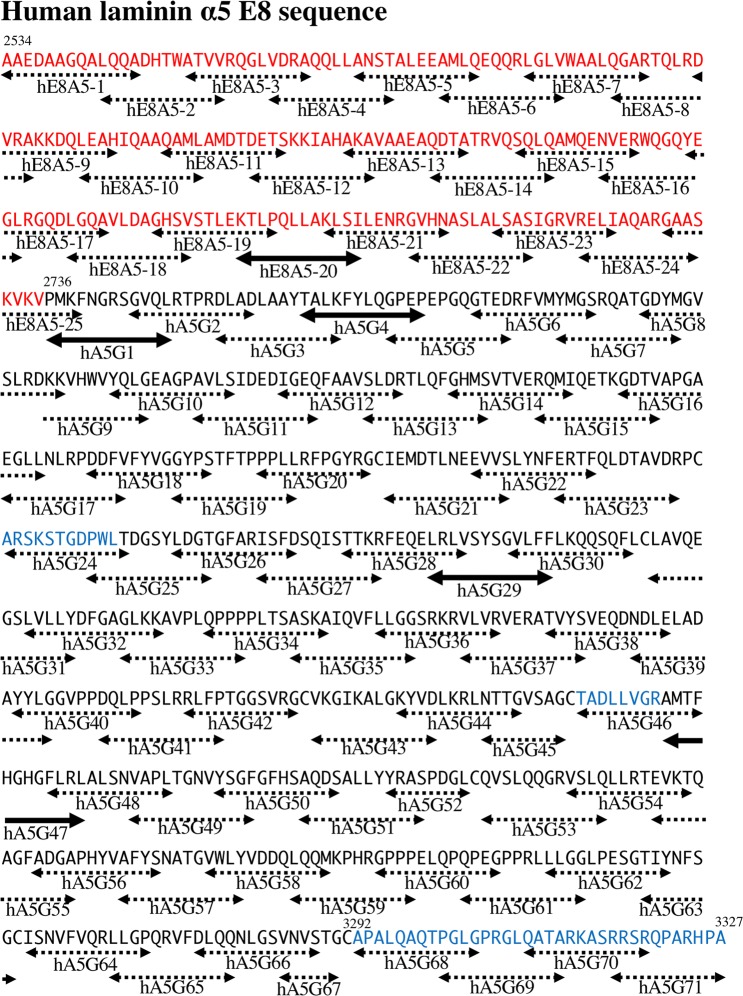
The amino acid sequence and peptides from the E8 region of human laminin α5. The sequence was derived from human laminin α5 (UniProtKB/Swiss-Prot, ID: O15230). Black, red, and blue letters represent LG module, LCC domain, and link region, respectively. The locations of peptides are indicated by two-way arrows. The active and inactive peptide sequences in the DG binding assay are denoted with bold and dotted arrows, respectively.

**Figure 5:**
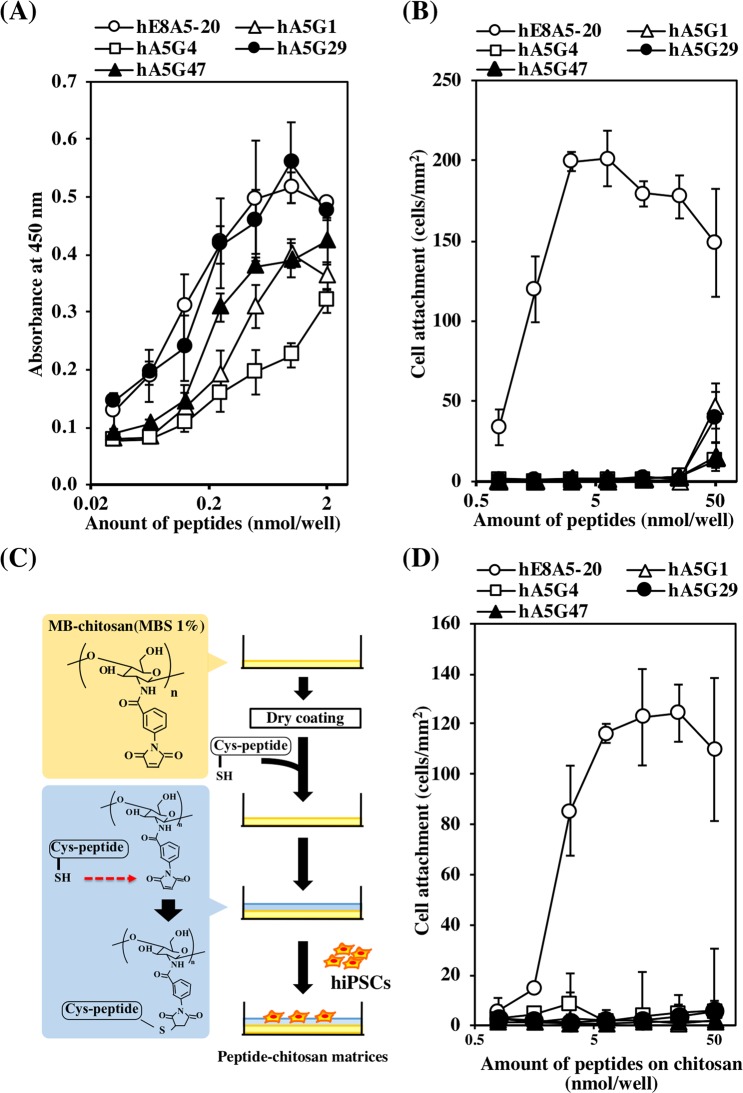
Characterization of DG binding peptides. (**A**) Dose dependent peptide binding to DG. Ninety six-well ELISA plates were coated with various amounts of peptides (hE8A5-20, hA5G1, hA5G4, hA5G29, and hA5G47). After blocking, the wells were incubated with MsDG-Fc at room temperature for 1 hour. Bound MsDG-Fc was detected with anti-human IgG Fc antibody. (**B**) Attachment of hiPSCs to the DG binding peptides. Ninety six-well ELISA plates were coated with various amounts of peptides and incubated with 201B7 cells. After incubation for 1 hour, adherent cells were stained and counted. The cells readily attached to hE8A5-20 but were significantly less adherent to the other peptides. (**C**) Preparation of peptide-conjugated chitosan. To conjugate the peptides, Cys was added at the N-termini. (**D**) Attachment of hiPSCs to the peptide-chitosan matrices. Various amounts of Cys-peptides were coupled to the MB-chitosan membranes in 96-well culture plates, as described in Methods. The plates were incubated with 201B7 cells. After incubation for 1 hour, adherent cells were quantified.

As shown above, the solid phase binding assay indicated that DG binding sites are localized on LM511-E8. Our previous study also showed that DG binds to two synthetic peptides derived from the laminin α2 chain LG4-5 modules^34^. In this study, we synthesized a series of peptides covering the amino acid sequences of LM511-E8 to identify DG binding sites (Figs 4 and S2–3). Peptides derived from the E8 region of laminin α5, β1 and γ1 chains were synthesized. The length of peptides were generally 12–13 amino acids, and they overlapped with neighbouring peptides by four amino acids. The glutamate or glutamic acid at the N-terminus of peptides form pyroglutamine^35^. To avoid the reaction, one amino acid was added at the N-terminus of glutamate or glutamic acid. In addition, to avoid the impact of disulphide bonds, cysteine residues were omitted. The series of peptides were dissolved in PBS (−), coated on 96-well ELISA plates, and tested for their DG binding activity. Of the peptides assayed, hE8A5-20, hA5G1, hA5-G4, hA5-G29, and hA5-G47 exhibited DG binding activity at 10 μg/mL (Figs 4 and S2). The series of peptides derived from laminin β1 and γ1 did not bind to DG (Figs S3 and S4). Moreover, we examined the dose-dependency of DG binding activity. hE8A5-20 and hA5G-29 derived from the laminin coiled-coil (LCC) domain and LG2 module, respectively, exhibited strong DG binding activity in a dose-dependent manner (Fig. 5A). The remaining active peptides revealed moderate DG binding activity dose-dependently.

### hiPSC attachment activity of DG binding peptides

We next examined if hiPSCs adhered to DG binding peptides using peptide-coated 96-well ELISA plates as well as solid phase binding assays. Of the five peptides, only hE8A5-20 exhibited high binding activity for attachment of hiPSCs (Fig. 5B). For culturing hiPSCs, cell adhesive peptides must be coated on cell culture dishes or plates. Although, coating of cell culture plates with the hE8A5-20 peptide posed considerable challenge, it did not exhibit cell attachment activity. Cell adhesive peptides often require mechanical support for cell culture. To address this issue, we have previously developed cell adhesive peptide-chitosan matrices for cell culture^36^. The peptide-chitosan matrices often improve cell adhesive activity of peptides. Chitosan matrices are natural polymers and have been used for medical applications, such as suture thread^37^. As shown in Fig. 5C, DG binding peptides were conjugated to chitosan-matrices. Of the five peptide-chitosan matrices, hE8A5-20-conjugated matrices exhibited activity for attachment of hiPSCs (Fig. 5D). Unfortunately, the activities of other peptides could not be improved on chitosan-matrices. Furthermore, although hiPSCs were cultured on hE8A5-20-conjugated matrices, the cells did not proliferate on the matrices (data not shown).

### The active core sequence of the hE8A5-20 peptide in DG binding activity

**Figure 6:**
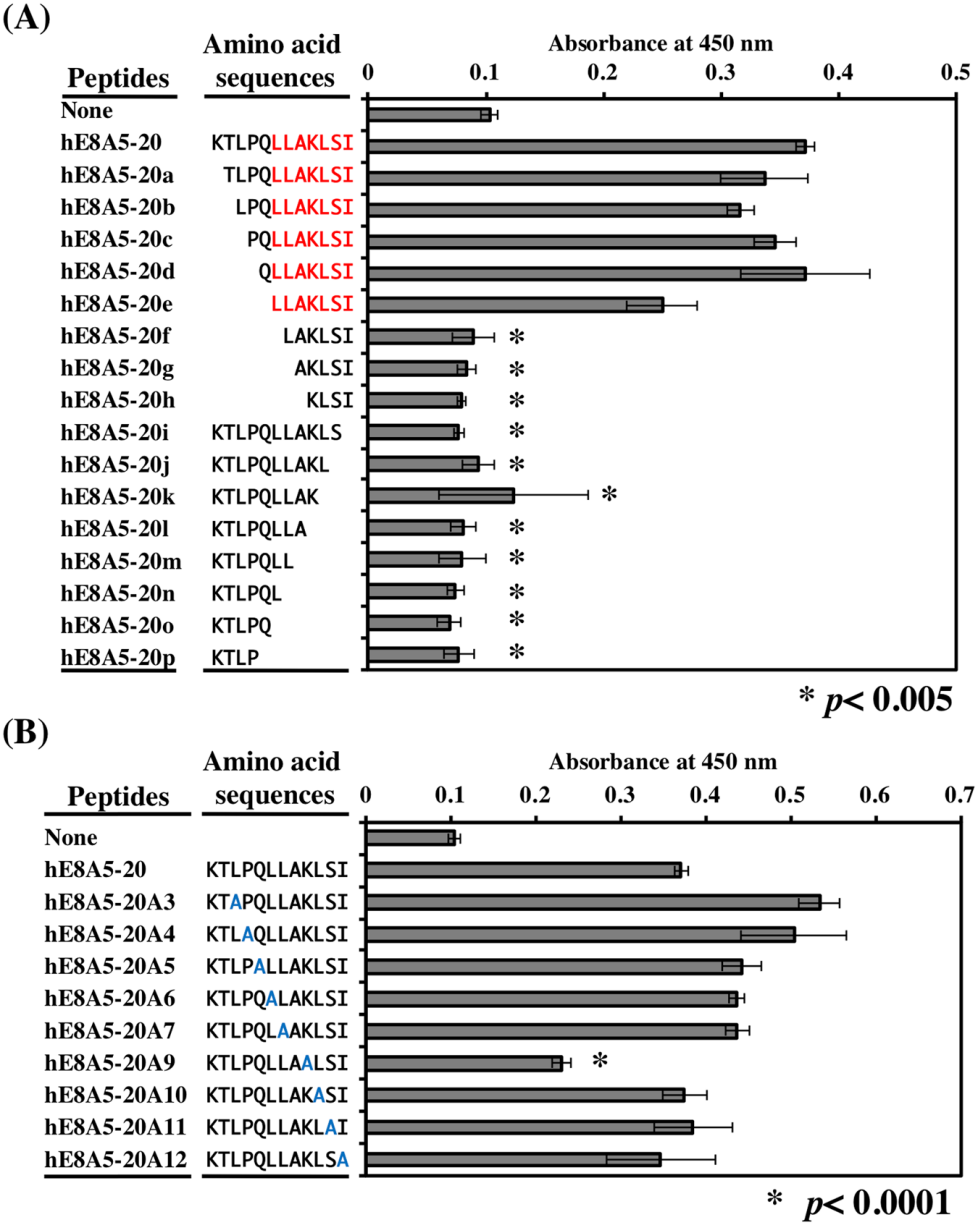
Identification of active amino acid sequences within the DG binding peptide. (**A**) Identification of the active core sequence of hE8A5-20. A series of hE8A5-20 truncations was used for the coating of 96-well ELISA plates. Each peptide was coated at a concentration of 1 nmol/well. Binding of MsDG-Fc to the peptides was assayed as described in Methods. Red letters represent the core active sequence. (**B**) Identification of the critical amino acid for DG binding. Alanine-substituted peptides were synthesized and used for solid phase binding assay. Blue letters represent the substituted alanines.

Because hE8A5-20 exhibited DG binding activity that mediates hiPSC attachment, the structural requirements were determined using systematically truncated N-terminal and C-terminal peptide derivatives of hE8A5-20 (Fig. 6A). hE8A5-20e (LLAKLSI), an N-terminal truncated peptide of hE8A5-20, retained activity, whereas hE8A5-20f (LAKLSI), with a deletion of the C-terminal leucine from hE8A5-20e, did not reveal activity. hE8A5-20i (KTLPQLLAKLS), with a deletion of the C-terminal isoleucine from hE8A5-20, abolished DG binding activity. These results show that the seven-amino acid sequence, LLAKLSI, is critical for hE8A5-20’s DG binding activity. Furthermore, we synthesized Ala-substituted hE8A5-20 peptides to identify crucial amino acid residues for DG binding activity (Fig. 6B). When the Lys at the 9th position was substituted with Ala, DG binding activity of hE8A5-20 was significantly decreased. Therefore, these results indicate that the 9th Lys is critical for hE8A5-20’s DG binding activity.

## Discussion

In this study, we characterised the adhesion of hiPSC to LM511-E8, which is generally used in stem cell culture. We first demonstrated the expression of DG, a non-integrin receptor, in hiPSCs cultured on either feeder cells or LM511-E8. HDFs, from which the hiPSCs originate, did not express DG, indicating that it was induced during cellular reprogramming. DG was originally identified in skeletal muscle as a component of the dystrophin-glycoprotein complex that interacts with the actin cytoskeleton and connects it to the muscle basement membrane^23^. DG is also expressed in various tissues and can interact with utrophin in non-muscle cells^38^. Because utrophin was expressed in hiPSCs (Fig. S4), it is likely that DG serves as a linker protein between utrophin and LM511-E8 in these cells.

The major DG binding sites of laminin (α5LG4-5 modules) are absent in LM511-E8. Therefore, the possibility of LM511-E8 binding to DG was not previously considered. In this study, although the overlay assay using LM511-E8 did not exhibit DG binding (data not shown), the solid phase binding assay indicated that LM511-E8 is capable of DG binding. When hiPSCs are cultured on whole LM511 with strong DG binding property, the self-renewal ability and pluripotency are maintained, similarly to when cultured on the E8 fragment only^3,7^. This suggests that because the attachment of hiPSCs to the E8 region mediated via integrin α6β1 is enough for maintaining stemness, the DG binding is not required for self-renewal ability and pluripotency of stem cells. However, because the function of DG-E8 binding is unclear in hiPSCs culture, it remains a possibility that the binding influences hiPSCs behaviour. Recently, Nguyen *et al*. demonstrated that whole laminin-511 with strong DG binding properties promote the endothelial cell differentiation of hESCs when induced with growth factors^39^. On the other hand, the E8 fragments with weak DG binding properties support the induction of cell differentiation, such as midbrain dopaminergic neurons and forebrain oligodendrocyte precursor cells from hiPSCs^40,41^. Thus, although the induction efficiency of cell differentiation is unclear between the whole molecule and E8 fragment of LM511, the difference of DG binding affinity may be one of cues in cell fate determination. Moreover, it is well known that differentiated cells appear at a low frequency in long-term stem cell cultures^7,42^. We cannot exclude a possibility that the DG binding influences the pluripotency of hiPSCs cultured on LM511-E8. To completely define the cell adhesion of hiPSCs to the substrata, a better strategy would be abolishing the binding activity of DG in LM511-E8.

DG is translated from a single gene followed by posttranslational cleavage, to give rise to the α and β subunits, the two non-covalently associated proteins^24^. α-DG, a heavily glycosylated protein, acts as a binding subunit for extracellular matrices, including laminins. It has a mucin-type *O*-glycosylation site in the central region of the molecule. The glucuronic acid and xylose (GlcA-Xyl) repeats in *O*-mannosyl glycans are required for laminin binding^43,44^. The laminin overlay assays suggest that DG of hiPSCs contains *O*-mannosyl glycans comprising GlcA-Xyl repeats. The *O*-mannosyl glycans are sequentially modified by several enzymes^45^. Of these, LARGE is a glycosyltransferase-like protein that generates polymers of GlcA-Xyl at the final DG modification step; these are required for the binding of DG to laminin. DG becomes functionally active when cellular reprogramming induces the biosynthetic pathway for *O*-mannosyl glycans.

**Figure 7:**
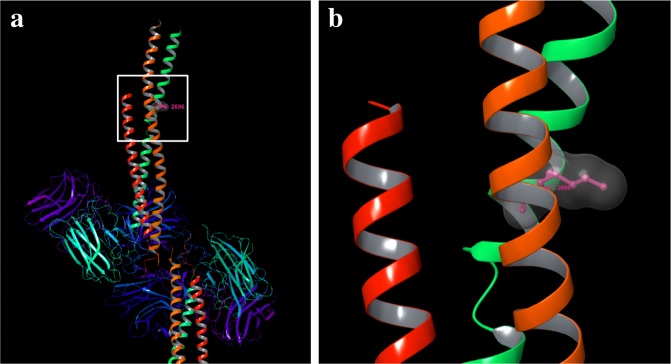
The amino acid residue critical for DG binding on a three-dimensional model of LM511-E8. Lys^2696^ was mapped onto the crystal structure of LM511-E8 (Protein Data Bank code 5XAU) (Takizawa *et al*., Sci Adv, 2017). The side chain of Lys faces outward in the α5LCC domain.

We have previously reported that it is possible to define the DG binding sequences within laminin using synthetic peptides, and to further identify the amino acids critical for DG binding^34^. In this study we identified five DG binding peptides in laminin α5, hE8A5-20, ^2688^KTLPQLLAKLSI^2699^; hA5G1, ^2736^PMKFNGRSGVQL^2747^; hA5G4, ^2760^TALKFYLQGPEP^2771^; hA5G29, ^2970^LRLVSYSGVLFF^2981^, and hA5G47, ^3124^AMTFHGHGFLRL^3135^. Previously, Wizemann *et al*. identified several basic amino acids important for α-DG binding through alanine substitution mutagenesis of the laminin α2 LG4-5 modules^46,47^. The five peptides contain Lys or Arg in their sequences. Recently, Takizawa *et al*. reported the crystal structure of LM511-E8 harbouring integrin α6β1 binding sites^48^. Integrin α6β1 binds to the bottom face of laminin α5LG1-3 modules with the laminin γ1-tail. We explored the location of DG binding sequences in the spatial structure of LM511-E8. ^2688^KTLPQLLAKLSI^2699^ of hE8A5-20 is located in the α-helix of the LCC domain. Because the side chain of K^2696^, critical for DG binding, faces outward (Fig. 7), it could interact with the carbohydrate chains of DG. DG binding sites are localized in laminin globular (LG) domains of laminins, agrin, perlecan, neurexin, pikachurin, and slit, which are currently known as DG ligands^32,49–51^. The DG binding site in the α5LCC domain that we identified, may provide new insight into the interaction of multiple receptors for laminin-511. The amino acid sequences of the remaining peptides are partially and fully buried in the α5LG1-3 modules. Proteolytic modification exposes cryptic sites with biological activity within larger molecules^52,53^. These DG binding sequences may also be cryptic sites that can be exposed under certain circumstances.

Although hE8A5-20-conjugated chitosan matrices exhibited adhesive activity for hiPSCs, they were unable to maintain cell growth. Because integrin α6β1 mediates potent cell attachment to LM511-E8, the self-renewal of hiPSCs would be mainly mediated through the receptor. Several groups reported that laminins with strong DG binding properties are key molecules for the differentiation of hESCs and hiPSCs^39,54,55^. However, it is still unclear as to how cell adhesion to laminins is involved in the commitment to differentiate. hE8A5-20-conjugated chitosan matrices may be useful to clarify the mechanism of cellular differentiation.

The development of transplantation therapy and drug discovery using hiPSCs requires modulation of cell differentiation. We found that functionally active DG is expressed in hiPSCs. Although the function of DG in stem cells is unclear, it is likely that the binding of DG mediates the differentiation of cells grown on laminins. We have produced the recombinant LM511-E8 that abolishes the binding activity of DG. The recombinant proteins will clarify the biological relevance of DG binding in hiPSC culture. Our approach also revealed that synthetic peptides mimic DG binding to laminin and mediate the adhesion of hiPSCs. The DG binding peptides and their amino acid sequences could be useful to design substrata for culturing differentiated cells, derived from hiPSCs, for cell-based therapy.

## Methods

### Reagents

Human recombinant laminin-111 (LM111), laminin-511 (LM511), and laminin-521 (LM521) were purchased from BioLamina (Sundbyberg, Sweden). iMatrix-511 (LM511-E8) and mouse EHS laminin (MsLM111) was from Nippi (Tokyo, Japan) and BD Biosciences (San Jose, CA), respectively.

### Cell culture

Two clones of hiPSCs, 201B7 and 454E2, were purchased from the RIKEN BioResource Center (Tsukuba, Japan). As described in Takahashi *et al*.^27^, the hiPSCs were maintained on feeder cells in Primate ES medium (ReproCELL, Yokohama, Japan), supplemented with basic fibroblast growth factor (bFGF, ReproCELL). For passaging of conventional colony cultures, hiPSCs were washed once with PBS (−), followed by incubation with Dissociation Solution for human ES/iPS Cells (ReproCELL), at 37 °C for 5 minutes. The detached colonies were collected and suspended in the growth medium. After a couple of passages, the hiPSCs were transferred to a feeder-free culture system, comprising LM511-E8^8^; culture dishes (Thermo Fisher Scientific, Waltham, MA) were coated with LM511-E8 (according to the manufacturer’s instructions) for preparing the feeder-free culture system. The colonies of hiPSCs were dissociated into single cells with cell dissociation buffer (Thermo Fisher Scientific). After incubation at 37 °C for 5 minutes, the cell dissociation buffer was removed. The dissociated cells were suspended in StemFit AK02 (ReproCELL) with Y-27632, and counted using LUNA Automated Cell Counter (Logos Biosystems, Gyeonggi-do, South Korea). About 6 × 10^4^ live cells were plated onto LM511-E8-coated 60 mm dishes; Y-27632 was used only at the time of plating^56^. The medium was replaced with StemFit AK02, without Y-27632, on the following day. The medium was changed every other day, until the cells reached 80–90% confluency. hiPSCs were subcultured every 6–8 days.

Human dermal fibroblasts (HDFs) were purchased from Cell Applications (San Diego, CA). The cells were maintained in DMEM containing 10% FBS, 0.1 mg/mL streptomycin and 100 U/mL penicillin G.

### Immunocytochemistry

For immunocytochemistry, hiPSCs were cultured on lumox multiwell plates (Sarstedt, Numbrecht, Germany) with feeder cells or coated with LM511-E8. After culturing for 4–7 days, the colonies of hiPSCs were doubly stained with the antibodies listed in Table 1. Briefly, the cells were fixed with 4% paraformaldehyde/PBS (−), and the unreacted aldehydes were blocked with 0.1 M Glycine/PBS (−). The fixed cells were permeabilized with 1% Triton-X100/PBS (−) and blocked in 10% normal goat serum. After blocking, the cells were incubated with primary antibodies. Secondary antibodies conjugated to Alexa 488 or 594 were used in these experiments. Hoechst 33258 was used for counter staining of the nuclei. After several washes, the cells were mounted in 90% glycerol, containing 0.1xPBS (−) and 1 mg/mL *p*-phenylenediamine. Images were captured using BZ-X700 (Keyence, Osaka, Japan).

**Table 1:** Primary antibodies.

Antibody to	Epitope	Clone	Host/Antigen Species	Source/Reference	Application
Dystroglycan	α-DG	VIA4	Mouse/Rabbit	DSHB, Iowa city, IA	ICC, FCM
Dystroglycan	α-DG (GlcA-Xyl)	IIH6	Mouse/Rabbit	DSHB, Iowa city, IA	ICC, FCM, IB
Nanog	Nanog peptide	—	Rabbit/Human	ReproCell, Yokohama, Japan	ICC
SSEA-4	SSEA-4	MC813-70	Mouse/Human	Merck, Kenilworth, NJ	FCM
TRA-1-81	TRA-1-81	TRA-1-81	Mouse/Human	Merck, Kenilworth, NJ	FCM
Laminin-111	mouse EHS laminn-111	—	Rabbit/Mouse	Sigma-Aldrich, St. Louis, MO	IB
Utrophin	C-terminus of utrophin	8A4	Mouse/Human	DSHB, Iowa city, IA	IB

IHC: immunocytochemistry; FCM, Flowcytometry; IB: immunoblotting.

### Flow cytometric analysis

Cells were detached with cell dissociation buffer (Thermo Fisher Scientific) and suspended in PBS (−) containing 0.1% BSA and 1 mM EDTA. The suspended cells were incubated with antibodies listed in Table 1. Following washing with PBS (−), cells were incubated with Alexa 488-labeled secondary antibody. The cells were then analyzed on a FACSCalibur flow cytometer (Becton Dickinson, San Jose, CA).

### DG preparation and immunoblotting

DG was enriched from hiPSC lysates. For preparation of cell lysates, hiPSCs were detached from culture dishes with the cell dissociation buffer and collected in tubes. They were lysed with lysis buffer (10 mM Tris–HCl, pH 7.5, 10 mM EDTA, 150 mM NaCl, 1% CHAPS) containing a protease inhibitor cocktail (Sigma-Aldrich, St Louis, MO). Lysates were clarified at 10,000 rpm to remove insoluble cell debris, and incubated with wheat germ agglutinin (WGA) agarose (Vector Laboratories, Burlingame, CA), at 4 °C overnight. The WGA complexes were washed with lysis buffer and mixed with SDS-PAGE sample buffer. Subsequently, the samples were separated on SDS-PAGE under reducing conditions and transferred to a PVDF membrane. DG, after being transferred on to the membrane, was probed with monoclonal antibody against α-DG (IIH6), followed by incubation with anti-mouse IgM antibody conjugated with horseradish peroxidase (Jackson ImmunoResearch, West Grove, PA). Bound antibodies were visualized with ECL detection regents (GE Healthcare).

### Laminin overlay assays

Laminin overlay assays were performed on PVDF membranes using mouse EHS laminin (MsLM111) and human laminin-511 (LM511), as previously described, with slight modifications^31^. Briefly, PVDF membranes were blocked with TBS (+) (10 mM Tris-HCl, 150 mM NaCl, 1 mM CaCl_2_, 1 mM MgCl_2_, pH7.5) containing 5% non-fat dry milk for 1 hour at room temperature. The membranes were incubated with 6 μg/mL of laminin at 4 °C overnight, in TBS (+) containing 3% BSA. The bound laminin was probed with anti-laminin-111 polyclonal antibody, followed by incubation with anti-rabbit IgG antibody conjugated with horseradish peroxidase. TBS (+) was used for antibody dilution and membrane washing.

### Preparation of conditioned media containing MsDG-Fc

cDNAs encoding mouse α-DG fused with human IgG_1_ Fc (MsDG-Fc) and control Fc expression vectors were constructed in our previous studies^18,32^. The recombinant proteins were expressed using the Expi293 Expression System according to the manufacturer’s instructions. The conditioned media (CM) were clarified through 0.22 μm pore filters and dialyzed against TBS (+). MsDG-Fc CM and Fc CM were diluted 1:3 with TBS (+) and used for solid phase binding assays.

### Solid phase binding assays

Solid phase binding assays were carried out with recombinant laminins coated onto high protein-binding capacity 96-well ELISA plates (IWAKI, Tokyo, Japan). Plates were blocked with PBS (−) containing 1% BSA and incubated with MsDG-Fc at room temperature for 1 hour. After washing with TBS (+), the bound MsDG-Fc was detected with a biotinylated anti-human IgG Fc antibody (Jackson ImmunoResearch). After further steps of washing, the bound antibodies were detected by addition of streptavidin-conjugated horseradish-peroxidase, followed by addition of 1 mg/mL *o*-phenylenediamine and 0.012% H_2_O_2_. The absorbance was measured at 450 nm with Multiskan GO microplate Spectrophotometer (Thermo Fisher Scientific).

### Synthetic peptides and cell attachment assays

The 9-fluorenylmethoxycarbonyl (Fmoc)-based solid-phase method with a C-terminal amide was used for the synthesis of all peptides, as described previously^57^. The purity and identity of the synthetic peptides were verified by analytical HPLC and electrospray ionization mass spectrometry at the Central Analysis Centre, Tokyo University of Pharmacy and Life Sciences. Cell attachment assays using synthetic peptides were performed as previously described with slight modifications^58^. Briefly, the synthetic peptides were dissolved in PBS (−) at 1 mM concentration, and 50 μL of this solution was added to each well of the high protein-binding capacity 96-well ELISA plates (IWAKI, Tokyo, Japan). After coating overnight at 4 °C, the plates were blocked with 1% BSA in PBS (−). The dissociated hiPSCs were suspended in 0.1% BSA in DMEM with Y-27632, plated at 1.0 × 10^4^ cells/50 μL/well. After incubation at 37 °C for 1 hour, the adhered cells were stained with Diff-Quik (International Reagents Corp., Kobe, Japan). The number of attached cells was counted under a microscope.

### Preparation of peptide chitosan matrices

Maleimidobenzoyloxy (MB)-chitosan was prepared as previously described^59^. For conjugation to chitosan membrane, a cysteine residue was added at the N-terminus, and two glycine residues were used as a spacer between the cysteine and the DG binding peptide sequences. Various amounts of peptides (0.5–50 nmol/well) in 1% trifluoroacetic acid (in Milli-Q water) and an equal amount of 1% NaHCO_3_ solution, were added into the wells and incubated for 2 hours. After conjugating the peptides, plates were washed three times with PBS (−) and blocked by the addition of 1% BSA in PBS (−) for 1 hour. The peptide chitosan matrices were used for cell attachment assays.

### Statistical analysis

Each bar or point indicates the mean of triplicate assays. Error bars represent standard deviation. Statistical significance was determined using Student’s *t* test.

## Supplementary information

Supplementary information
