# Supplementary material for: Characterization of dystroglycan binding in adhesion of human induced pluripotent stem cells to laminin-511 E8 fragment
Source: Sci Rep. 2019 Sep 10;9:13037. doi: 10.1038/s41598-019-49669-x (PMC6737067; doi:10.1038/s41598-019-49669-x)
Supplement: Supplementary file 1 — Supplementary information [file 41598_2019_49669_MOESM1_ESM.pdf]

Supplementary information

Characterization of dystroglycan binding in adhesion of human induced pluripotent stem cells to laminin-511 E8 fragment

Yumika Sugawara [1], Keisuke Hamada [1], Yuji Yamada [1], Jun Kumai [1], Motoi Kanagawa [2], Kazuhiro Kobayashi [2], Tatsushi Toda [3], Yoichi Negishi [4], Fumihiko Katagiri [1], Kentaro Hozumi [1], Motoyoshi Nomizu [1], and Yamato Kikkawa [1] *

[1] Department of Clinical Biochemistry, Tokyo University of Pharmacy and Life Sciences, Tokyo 192-0392, Japan.
[2] Division of Molecular Brain Science, Kobe University Graduate School of Medicine, Kobe, Hyogo 650-0017
[3] Department of Neurology, Graduate School of Medicine, The University of Tokyo, Tokyo 113-0033
[4] Department of Drug Delivery and Molecular Biopharmaceutics, Tokyo University of Pharmacy and Life Sciences, Tokyo 192-0392

*Corresponding author

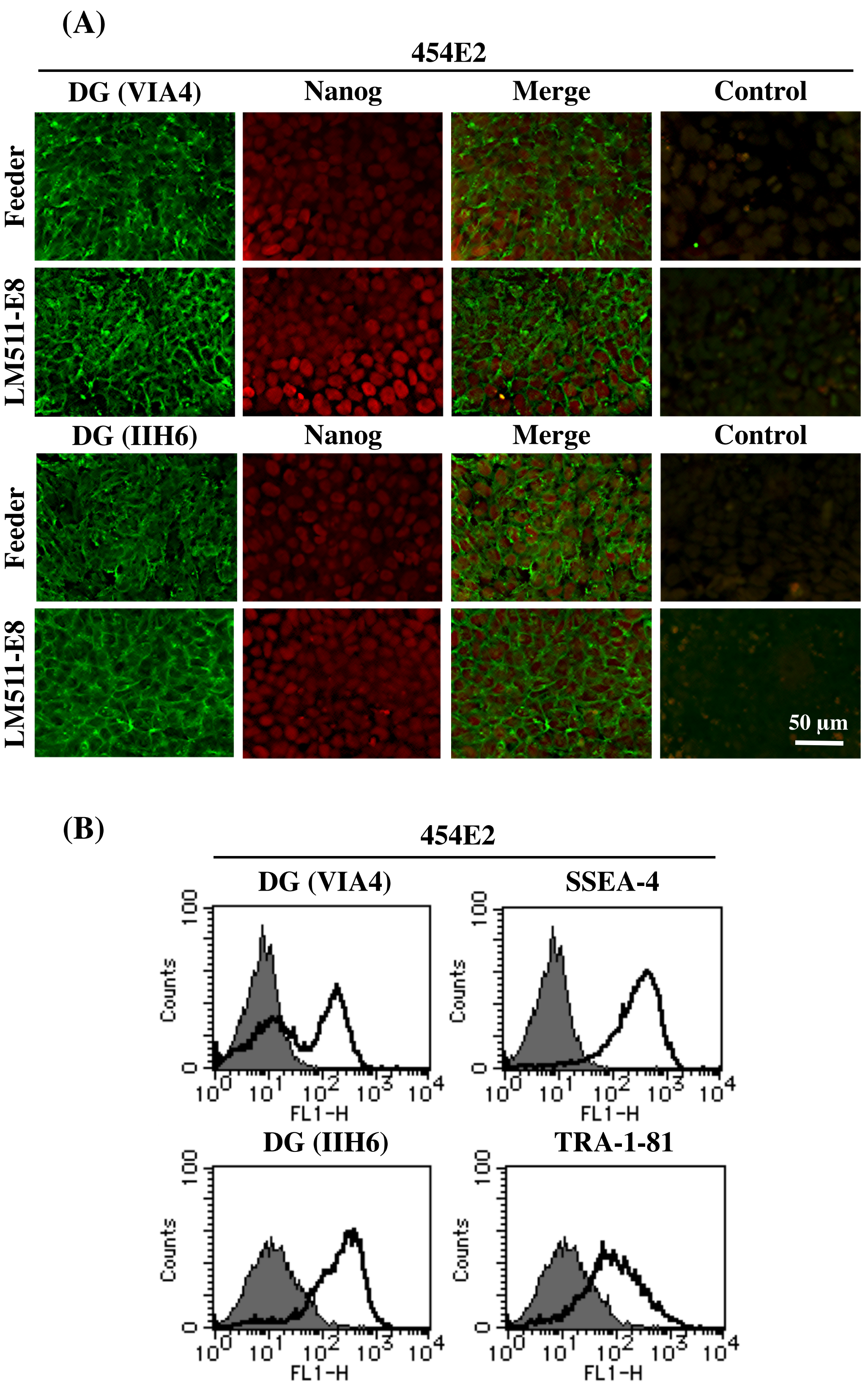

Figure S1. DG expression in 454E2 cells. (A) Immunostaining for DG and Nanog in hiPSCs cultured on feeder cells or LM511-E8. Colonies were doubly stained with antibodies (VIA4 and IIH6) to DG (green) and Nanog (red). Images of DG and Nanog staining are merged. Control was without primary antibodies. (B) Flow cytometric analysis of expression of DG and two stem cell markers in hiPSCs. The expression of DG (VIA4

and IIH6), SSEA-4, and TRA-1-81 is shown as a solid line. Gray fill indicates control IgG (mouse IgG, upper panel; mouse IgM lower panel). 454E2 cells highly express DG, similar to 201B7.

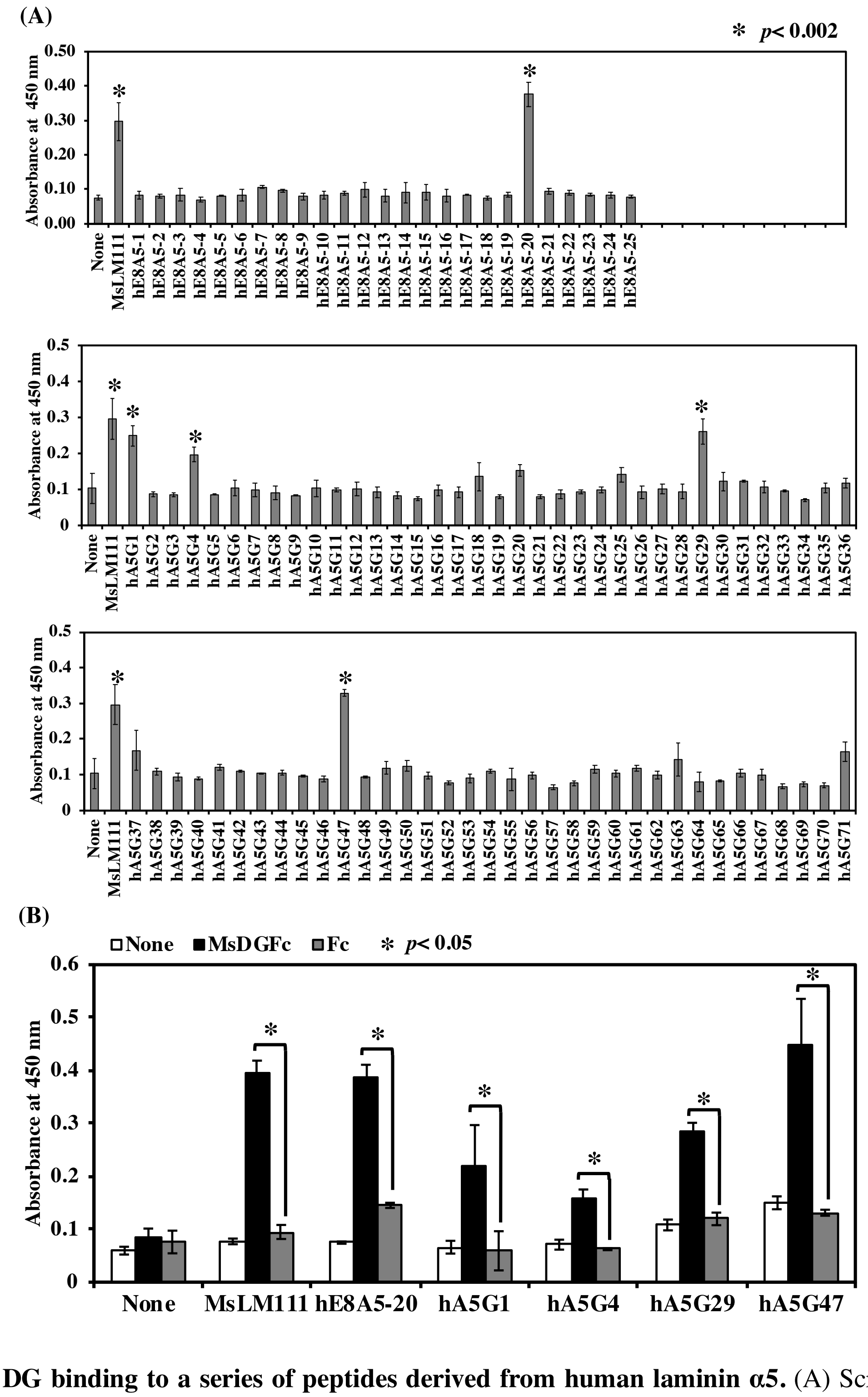

Figure S2. DG binding to a series of peptides derived from human laminin α5. (A) Screening of DG binding peptides. Ninety six-well ELISA plates were coated with a series of synthetic peptides derived from human laminin α5 E8 sequence (10 μg/mL). After blocking, the wells were incubated with conditioned media containing MsDG-Fc at room temperature for 1 hour. MsLM111 (20 nM) was used as positive control. Bound MsDG-Fc was detected with anti-human IgG Fc antibody. (B) The influence of Fc tag on MsDG-Fc binding to peptides. Ninety six-well ELISA plates were coated with hE8A5-20, hA5G1, hA5G4, hA5G29, and hA5G47 peptides. After blocking, the wells were incubated with conditioned media containing MsDG-Fc or Fc at room

temperature for 1 hour. DG binding assays were performed as described in Methods. The Fc tag was not responsible for DG binding.

(A)

Human laminin β1 LCC domain E8 sequence

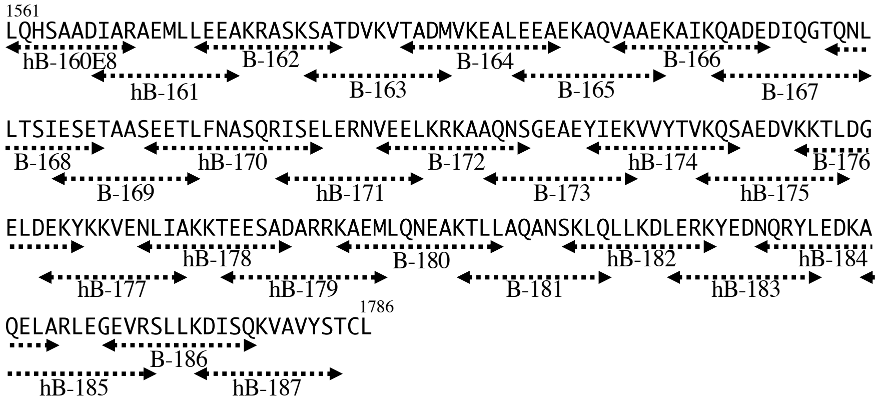

Human laminin γ1 LCC domain E8 sequence

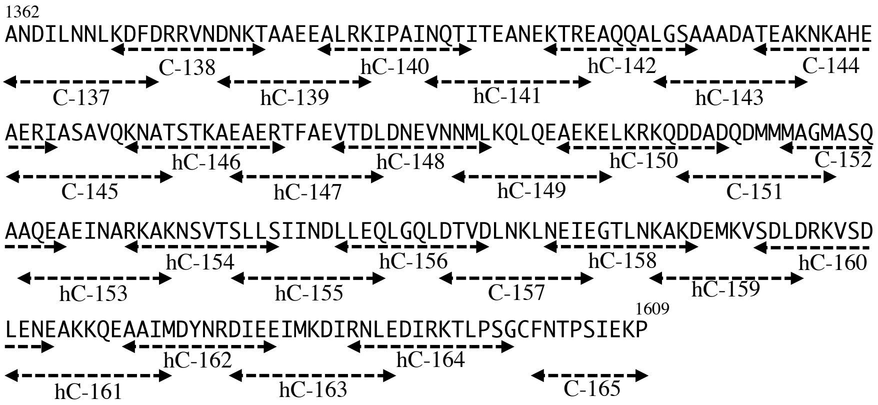

(B)

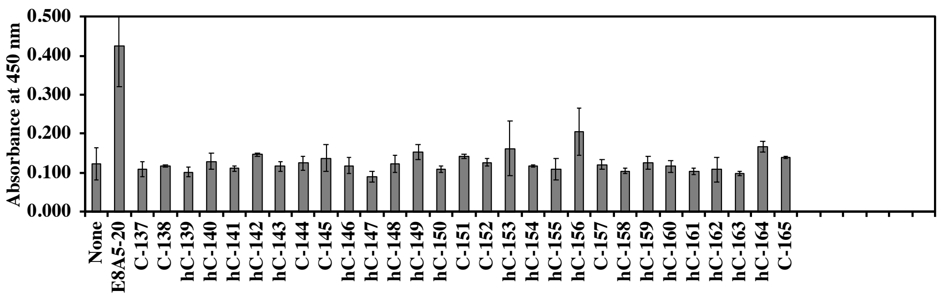

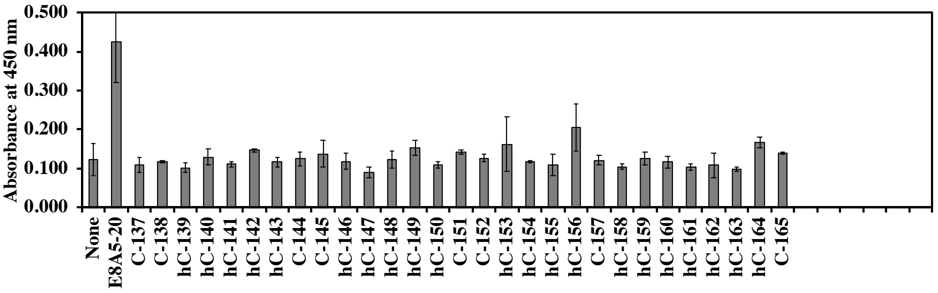

Figure S3. DG binding to a series of peptides derived from human laminin β1 and γ1 chains. (A) The amino acid sequence and peptides from the E8 region of human laminin β1 and γ1 chains. The sequences were derived from human laminin β1 (UniProtKB/Swiss-Prot, ID: P07942) and γ1 (UniProtKB/Swiss-Prot, ID: P11047) chains. The locations of peptides are indicated by dotted two-way arrows. (B) Screening of DG

binding peptides. Binding of MsDG-Fc to the peptides was assayed as described in Methods. E8A5-20 was used as positive control. No peptide bound to DG.

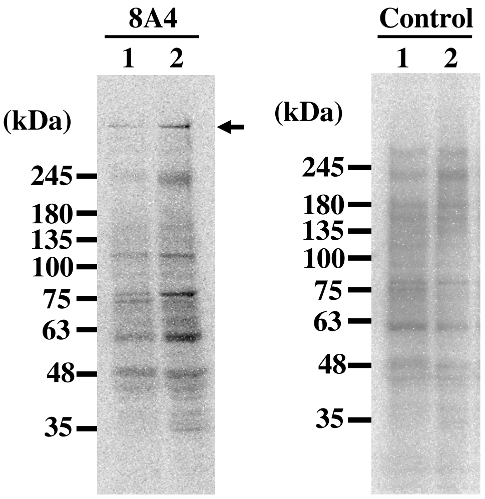

Figure S4. Utrophin expression in hiPSCs. Lysates prepared from 201B7 (lane 1) and 454E2 (lane 2) cells were separated on a 5-20% gradient gel under reducing conditions, and immunoblotted with anti-utrophin monoclonal antibody (8A4, left panel). Control is without primary antibody (right panel). Although unspecific signals of secondary antibody appeared below 250 kDa, utrophin migrated at the proper size (400 kDa) in both cells. Arrow indicates the bands of utrophin.
